# Supplementary material for: Accidental hypothermia in emergency care: multifactorial triage-based prediction of early critical outcomes in a temperate-climate cohort
Source: PLoS One. 2025 Oct 9;20(10):e0334328. doi: 10.1371/journal.pone.0334328 (PMC12510580; doi:10.1371/journal.pone.0334328)
Supplement: S1 Table — Examples of chief complaints, primary and secondary modifiers by triage category [11, 12]. (PDF) [file pone.0334328.s001.pdf]

S1 Table

MSTR category	Chief complaints (automatically place the patient in this category)	Primary modifiers (vital signs, clinical findings)	Laboratory & secondary modifiers
**MSTR I (Resuscitation)**	- Cardiac / respiratory arrest<br>- Continuous generalized seizure<br>- Complete airway obstruction / apnoea / periarrest breathing<br>- Major trauma with shock signs<br>- Umbilical-cord or fetal-part prolapse (≥ 20 weeks)<br>- Massive vaginal bleed (3rd trimester)	- Glasgow Coma Scale ≤ 8<br>- SpO_2 <90 %<br>- Any HR, RR or SBP in CTAS/MSTR red zone (e.g. adult HR > 130/min or <40/min; RR > 35/min or < 8/min; SBP < 80 mmHg)<br>- Signs of shock: capillary-refill > 3 s, cold/mottled skin, weak/absent pulse<br>- Extreme work of breathing (one-word sentences, cyanosis, silent chest)	No isolated lab value promotes straight to MSTR I
**MSTR II (Emergent)**	- Chemical eye burn<br>- Palpitations/arrhythmia with prior CPR<br>- Syncope/presyncope without prodrome<br>- Burns > 25 % TBSA<br>- Frostbite with pulseless limb<br>- Traumatic amputation<br>- Cardiac-origin chest pain<br>- Stroke signs <4 h 30 min<br>- Marked stridor + drooling/dysphagia<br>- Pregnancy > 20 weeks: contractions ≤ 2 minutes, absent fetal movement/heartbeat, or eclampsia<br>- Violent/homicidal behavior or active self-harm attempt	- GCS 9–12<br>- SpO_2 90–92 %<br>- HR, RR, SBP in orange zone<br>- Core T > 40 °C or <32 °C<br>- Pain 7–10/10<br>- PEFR <40 % predicted<br>- Moderate–severe respiratory distress<br>- Moderate dehydration	- Blood glucose <3 or > 18 mmol/L<br>- Hypertensive crisis ≥ 220/130 mm Hg with symptoms<br>- SIRS/sepsis screen: core T > 38 °C or <36 °C plus ≥ 2 of HR > 90, RR > 20 or $PaCO_2$ <32 mm Hg, WBC > 12 000/ <4 000/ > 10 % bands
**MSTR III (Urgent)**	- Positional dizziness (no focal deficit)<br>- Limb injury with neuro-vascular deficit or tight cast<br>- High-energy blunt trauma pending spinal clearance<br>- Low-risk blood/body-fluid exposure<br>- Pregnancy > 20 weeks: contractions > 2 minutes or ruptured membranes<br>- Core T 32–35 °C	- GCS 13–14<br>- SpO_2 92–94 %<br>- HR, RR, SBP in yellow zone<br>- Fever > 38.5 °C and ill-appearing child (3 months – 3 years) or infant <3 months with T > 38 °C / <36 °C<br>- Pain 4–6/10<br>- PEFR 40–60 %<br>- Mild–moderate respiratory distress<br>- Mild–moderate dehydration	- Hypertension ≥ 220/130 mm Hg without symptoms or 200–220 / 110–130 mm Hg
**MSTR IV (Less Urgent)**	- Localized cellulitis / hot, red limb<br>- Minor laceration requiring suture<br>- Small gastrointestinal or rectal bleed<br>- Noninjury sexual assault > 12 h<br>- Stable dementia-related confusion<br>- Mild urinary-tract symptoms<br>- Constipation with pain <4/10<br>- Core T > 35 °C + normal vitals<br>- Fever > 38.5 °C in child > 3 years, appears well	- GCS 15<br>- SpO_2 > 94 %<br>- HR, RR, SBP age-normal<br>- Pain 1–3/10	No lab trigger beyond this category
**MSTR V (Non-urgent)**	- Nasal congestion/allergic rhinitis<br>- Result review / prescription renewal<br>- Asymptomatic infectious contact<br>- Gradual hearing loss	- All vitals normal for age<br>- Pain 0–2/10	No relevant laboratory trigger

GCS – Glasgow Coma Scale, **HR** – heart rate, **RR** – respiratory rate, **SBP** – systolic blood pressure, **SpO_2** – peripheral oxygen saturation, **PEFR** – peak expiratory flow rate, **TBSA** – total body surface area, **SIRS** – systemic inflammatory response syndrome, **WBC** – white blood cell count, **$PaCO_2$** – partial pressure of carbon dioxide.
