# Supplementary material for: Accidental hypothermia in emergency care: multifactorial triage-based prediction of early critical outcomes in a temperate-climate cohort
Source: PLoS One. 2025 Oct 9;20(10):e0334328. doi: 10.1371/journal.pone.0334328 (PMC12510580; doi:10.1371/journal.pone.0334328)
Supplement: S2 Table — (PDF) [file pone.0334328.s002.pdf]

S2 Table

Parameter	Threshold → MSTR category
**Oxygen saturation**	- <90 % → MSTR I<br>- 90–92 % → MSTR II<br>- 92–94 % → MSTR III<br>- > 94 % → MSTR IV/V
**Peak expiratory flow**	- <40 % predicted → MSTR II<br>- 40–60 % → MSTR III
**Core temperature**	- > 40 °C → MSTR II<br>- 32–35 °C → MSTR III<br>- <32 °C → MSTR II<br>- Infant 0–3 months: > 38 °C or < 36 °C → MSTR II<br>- Immunocompromised any age: > 38 °C or < 36 °C → MSTR II<br>- Child > 3 years: > 38.5 °C + appears ill → MSTR III<br>- Child > 3 years: > 38.5 °C+ appears well → MSTR IV
**Blood glucose**	<3 or > 18 mmol/L→ MSTR II
**Blood pressure (adult)**	- ≥ 220/130 mm Hg with symptoms → MSTR II<br>- ≥ 220/130 mm Hg no symptoms or 200–220/110–130 mm Hg → MSTR III
**Pain score**	- 7–10 /10 → MSTR II<br>- 4–6 /10 → MSTR III<br>- 1–3 /10 → MSTR IV<br>- 0–2 /10 → MSTR V
**Dehydration**	- Severe with shock → MSTR I<br>- Moderate → MSTR II<br>- Mild → MSTR III<br>- Possible → MSTR IV
**SIRS / Sepsis screen**	Core T > 38 °C or <36 °C plus ≥ 2 of: HR > 90/min RR > 20 min or $PaCO_2$ <32 mm Hg; WBC > 12 000 / < 4 000 / > 10 % bands → minimum MSTR II
**Environmental hypothermia**	- Core T <32 °C → MSTR II<br>- 32–35 °C → MSTR III

HR – heart rate, **RR** – respiratory rate, **SBP** – systolic blood pressure, **SpO_2** – peripheral oxygen saturation, **$PaCO_2$** – partial pressure of carbon dioxide, **WBC** – white blood cell count, **SIRS** – systemic inflammatory response syndrome, **Core T** – core temperature.
