# Supplementary material for: Accidental hypothermia in emergency care: multifactorial triage-based prediction of early critical outcomes in a temperate-climate cohort
Source: PLoS One. 2025 Oct 9;20(10):e0334328. doi: 10.1371/journal.pone.0334328 (PMC12510580; doi:10.1371/journal.pone.0334328)
Supplement: S3 Table — (PDF) [file pone.0334328.s003.pdf]

S3 Table

Parameter	Specification (°C)
Displayed measurement range	20 – 42.2
Accuracy 35.0 – 42.0 °C	±0.2
Accuracy < 35.0 °C or > 42.0 °C	±0.3
Operating ambient range	10 – 40
Measurement time	2 – 3 s
ASTM compliance	E 1965-98
