# Supplementary material for: Accidental hypothermia in emergency care: multifactorial triage-based prediction of early critical outcomes in a temperate-climate cohort
Source: PLoS One. 2025 Oct 9;20(10):e0334328. doi: 10.1371/journal.pone.0334328 (PMC12510580; doi:10.1371/journal.pone.0334328)
Supplement: S4 Table — (PDF) [file pone.0334328.s004.pdf]

# S4 Table

| Admission_month | Monthly mean outdoor temperature °C |
|---|---|
| 2020-01 | 0.9 |
| 2020-02 | 7.3 |
| 2020-03 | 9.1 |
| 2020-05 | 16.4 |
| 2020-10 | 12.9 |
| 2020-11 | 6.5 |
| 2020-12 | 4.4 |
| 2021-01 | 2.8 |
| 2021-02 | 4.1 |
| 2021-03 | 7.6 |
| 2021-04 | 9.7 |
| 2021-05 | 15.2 |
| 2021-11 | 7.1 |
| 2021-12 | 2.7 |
| 2022-01 | 3.0 |
| 2022-03 | 7.3 |
| 2022-04 | 10.9 |
| 2022-09 | 17.0 |
| 2022-10 | 14.2 |
| 2022-11 | 7.7 |
| 2022-12 | 3.3 |
| 2023-01 | 5.1 |
| 2023-02 | 5.1 |
| 2023-03 | 9.2 |
| 2023-04 | 11.2 |
| 2023-06 | 21.5 |
| 2023-11 | 7.6 |
| 2023-12 | 4.0 |
| 2024-01 | 3.3 |
| 2024-02 | 10.2 |
| 2024-03 | 11.2 |
| 2024-04 | 14.9 |
| 2024-07 | 26.6 |
| 2024-09 | 19.8 |
| 2024-10 | 13.6 |
| 2024-11 | 5.6 |
| 2024-12 | 3.9 |
