# Supplementary material for: Accidental hypothermia in emergency care: multifactorial triage-based prediction of early critical outcomes in a temperate-climate cohort
Source: PLoS One. 2025 Oct 9;20(10):e0334328. doi: 10.1371/journal.pone.0334328 (PMC12510580; doi:10.1371/journal.pone.0334328)
Supplement: S5 Table — The triage category and the tympanic temperature on admission are independently significant predictors of critical outcome. In the combined model, both retain their effect, while collinearity is not present. Model-level discrimination (AUC) improves from 0.683 to 0.740 when moving from triage to triage + temperature, and explanatory power (Nagelkerke R²) also increases. (PDF) [file pone.0334328.s005.pdf]

S5 Table

Predictor	OR	CI_low	CI_high	p_value	AUC	Nagelkerke_R2	N
Triage category	0.31	0.157	0.61	< 0.001	0.683	0.146	131
Admission temperature (°C)	0.828	0.738	0.928	0.001	0.666	0.116	131
Swiss staging (per category)	1.877	1.208	2.919	0.005	0.644	0.086	131
WMS classification (per category)	2.035	1.203	3.442	0.008	0.637	0.078	131

Model	OR_Triage	CI_Triage_low	CI_Triage_high	p_Triage	Pair	OR_Pair	CI_Pair_low	CI_Pair_high	p_Pair	VIF_Triage	VIF_Pair	AUC	Nagelkerke_R2	N
Triage + temperature	0.39	0.197	0.774	0.007	Temperature	0.866	0.768	0.976	0.019	1.075	1.075	0.74	0.199	131
Triage + Swiss staging	0.372	0.189	0.733	0.004	Swiss	1.598	1.002	2.549	0.049	1.051	1.051	0.728	0.183	131
Triage + WMS classification	0.363	0.185	0.71	0.003	WMS	1.748	1.003	3.047	0.049	1.033	1.033	0.726	0.183	131
