# Supplementary material for: Accidental hypothermia in emergency care: multifactorial triage-based prediction of early critical outcomes in a temperate-climate cohort
Source: PLoS One. 2025 Oct 9;20(10):e0334328. doi: 10.1371/journal.pone.0334328 (PMC12510580; doi:10.1371/journal.pone.0334328)
Supplement: S6 Table — Discrimination indices (AUC, sensitivity, specificity, likelihood ratios, predictive values) are shown at the Youden-optimal threshold for each predictor. Calibration indices (Brier score, intercept, slope) refer only to the triage + temperature model, which achieved the highest AUC value among the tested predictors. Abbreviations: AUC, area under the ROC curve; LR, likelihood ratio; PPV, positive predictive value; NPV, negative predictive value. (PDF) [file pone.0334328.s007.pdf]

S6 Table

Predictor	AUC	AUC_CI_low	AUC_CI_high	Sensitivity	Specificity	LR +	LR -	PPV	NPV	Decision threshold	N
Triage category (MSTR)	0.683	0.598	0.767	0.705	0.655	2.043	0.451	0.508	0.814	0.49	131
Admission temperature (°C)	0.666	0.566	0.764	0.523	0.747	2.067	0.639	0.511	0.756	0.376	131
Swiss staging	0.644	0.545	0.736	0.523	0.724	1.895	0.659	0.489	0.75	0.442	131
WMS classification	0.637	0.536	0.73	0.523	0.724	1.895	0.659	0.489	0.75	0.471	131
Triage + temperature (logistic score)	0.74	0.644	0.829	0.795	0.621	2.097	0.33	0.515	0.857	0.282	131

Metric	Value
Event prevalence	0.336
Brier (apparent)	0.188
Brier (null, prevalence)	0.223
Brier (optimism-corrected)	0.198
Calibration intercept (apparent)	0.000
Calibration slope (apparent)	0.999
Calibration slope (optimism-corrected)	0.981
