# Supplementary material for: Accidental hypothermia in emergency care: multifactorial triage-based prediction of early critical outcomes in a temperate-climate cohort
Source: PLoS One. 2025 Oct 9;20(10):e0334328. doi: 10.1371/journal.pone.0334328 (PMC12510580; doi:10.1371/journal.pone.0334328)
Supplement: S7 Table — There is a moderate but low-risk relationship between triage and tympanic temperature, collinearity is not confirmed. (PDF) [file pone.0334328.s008.pdf]

S7 Table

Metric	Value
Pearson r (triage vs temperature)	0.265
Pearson p-value	0.002
Spearman rho (triage vs temperature)	0.309
Spearman p-value	< 0.001
VIF (triage; model triage+temp)	1.075
VIF (temp; model triage+temp)	1.075
Condition index (max, triage<->temp)	1.311
