# Supplementary material for: Accidental hypothermia in emergency care: multifactorial triage-based prediction of early critical outcomes in a temperate-climate cohort
Source: PLoS One. 2025 Oct 9;20(10):e0334328. doi: 10.1371/journal.pone.0334328 (PMC12510580; doi:10.1371/journal.pone.0334328)
Supplement: Attachment — Submitted filename: cover_letter_tech.docx [file pone.0334328.s009.docx]

**Dear Editorial Office,**

We have addressed all requested points at the pre-review technical check:

1. Completed Human Participants Research Checklist with line references.
2. Uploaded original Hungarian ethics approval and a brief English translation.

We hope the submission is now compliant and look forward to peer review.

Kind regards,

Kornél Ádám

Corresponding author
